# Supplementary material for: Stress Responsiveness and Emotional Eating Depend on Youngsters’ Chronic Stress Level and Overweight
Source: Nutrients. 2021 Oct 19;13(10):3654. doi: 10.3390/nu13103654 (PMC8540677; doi:10.3390/nu13103654)
Supplement: Supplementary file 1 [file nutrients-13-03654-s001.zip › Figure S1.pdf]

Figure S1. Repeated measures ANOVA with time as within-subject factor

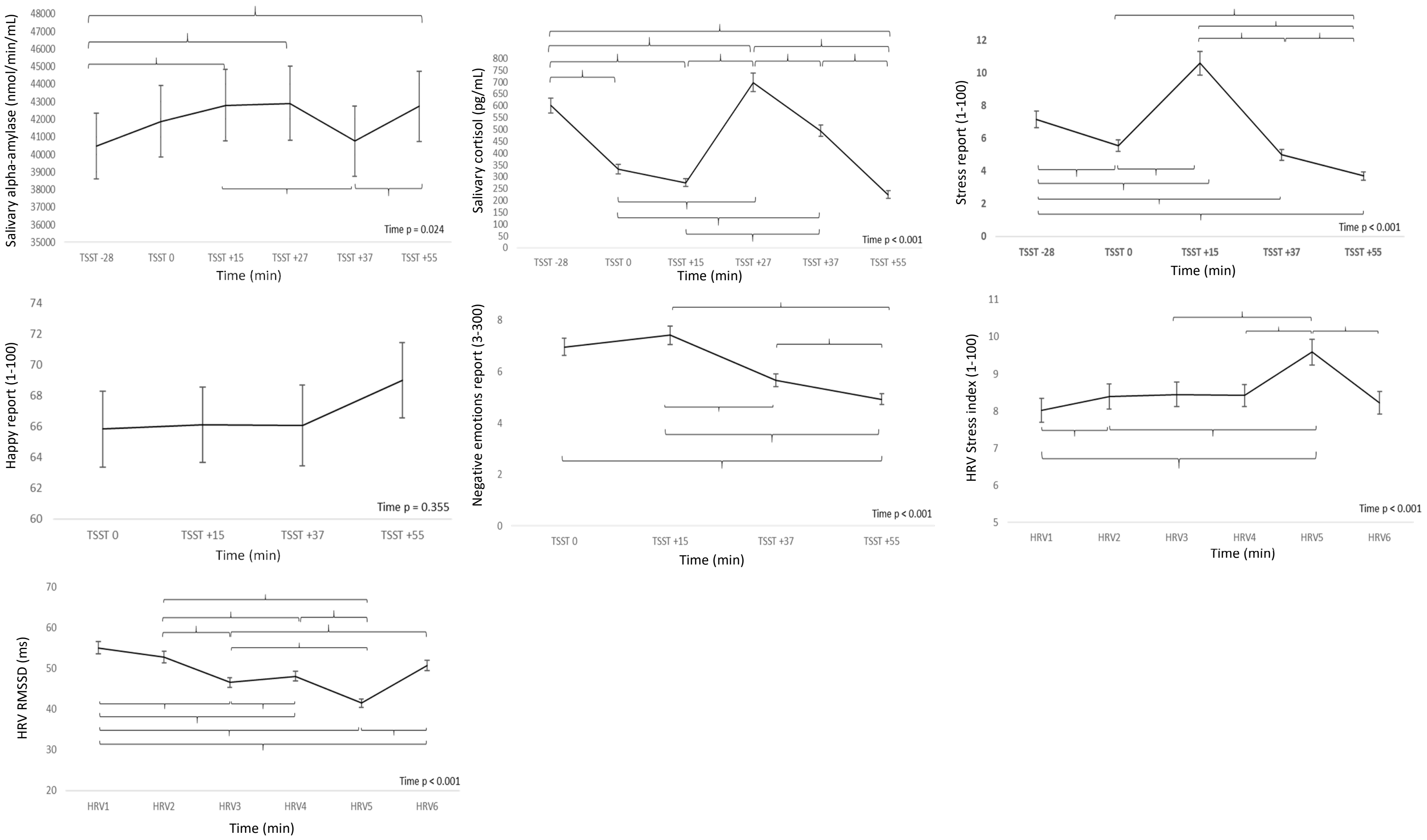

RMSSD: Root Mean Square of the Successive Differences; HRV: heart rate variability. HRV1 and HRV2 were taken before the Trier social stress test for children (TSST-C), HRV3 during the TSST-C, HRV4 between the TSST-C and the food lab, HRV5 during the food lab and HRV6 after the food lab. Horizontal brackets show significant changes ($p \leq 0.05$) over the different time points.
