# Supplementary material for: Stress Responsiveness and Emotional Eating Depend on Youngsters’ Chronic Stress Level and Overweight
Source: Nutrients. 2021 Oct 19;13(10):3654. doi: 10.3390/nu13103654 (PMC8540677; doi:10.3390/nu13103654)
Supplement: Supplementary file 1 [file nutrients-13-03654-s001.zip › Figure S2.pdf]

Figure S2. Group differences in stress reactivity/recovery by repeated measures ANOVA.

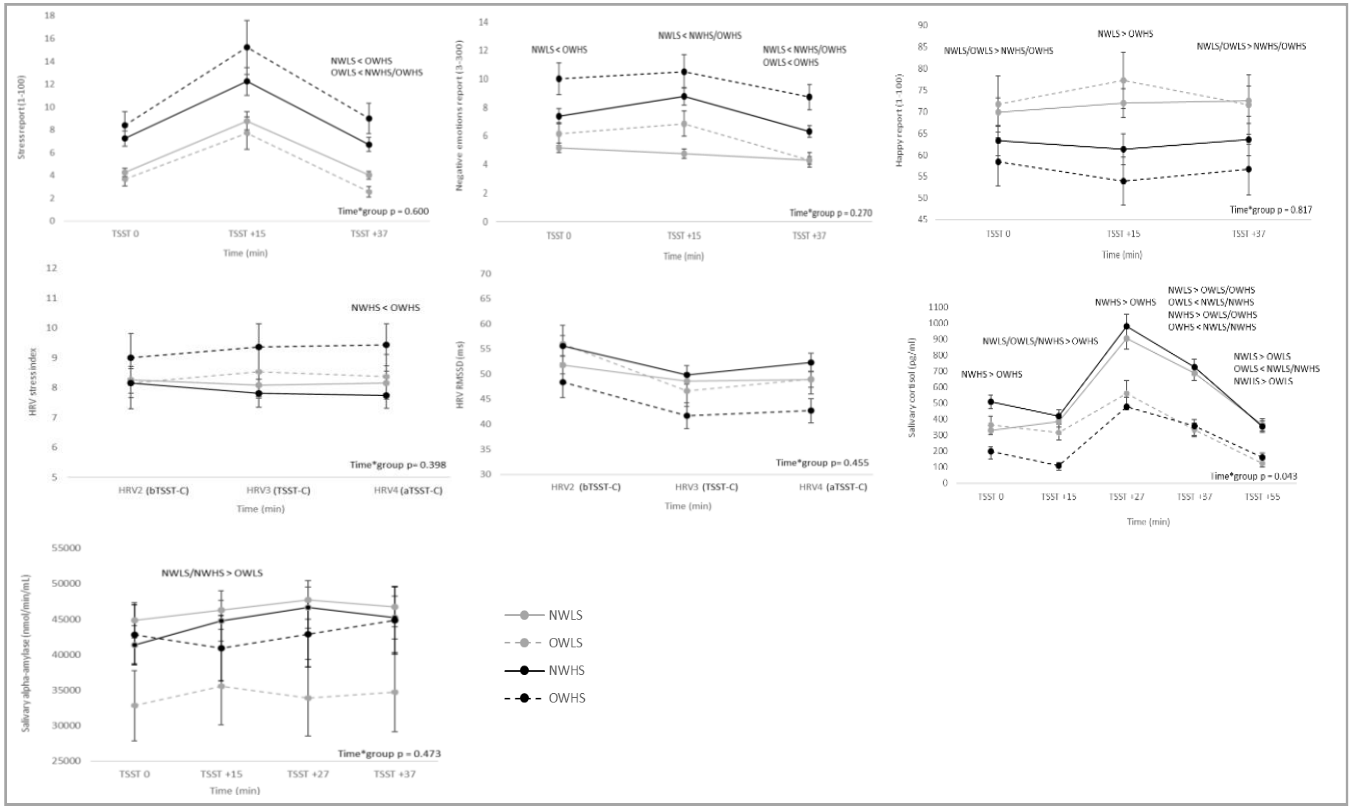

Differences (estimated marginal means and standard error) in stress reactivity/recovery between the four groups: Normal Weight Low Stress (NWLS), Overweight Low Stress (OWLS), Normal Weight High Stress (NWHS) and Overweight High Stress (OWHS) adolescents. Significant group differences (p≤0.05) are visualized per stress parameter at the corresponding timepoint/interval. TSST-C: Trier Social Stress Test – Children; bTSST-C: before TSST-C; aTSST-C: after TSST-C; RMSSD: Root Mean Square of the Successive Differences.
