# Supplementary material for: Stress Responsiveness and Emotional Eating Depend on Youngsters’ Chronic Stress Level and Overweight
Source: Nutrients. 2021 Oct 19;13(10):3654. doi: 10.3390/nu13103654 (PMC8540677; doi:10.3390/nu13103654)
Supplement: Supplementary file 1 [file nutrients-13-03654-s001.zip › Figure S3.pdf]

Figure S3: Moderating effects of stress measures: reactivity, recovery or AUCi on associations between chronic stress weight groups and food intake

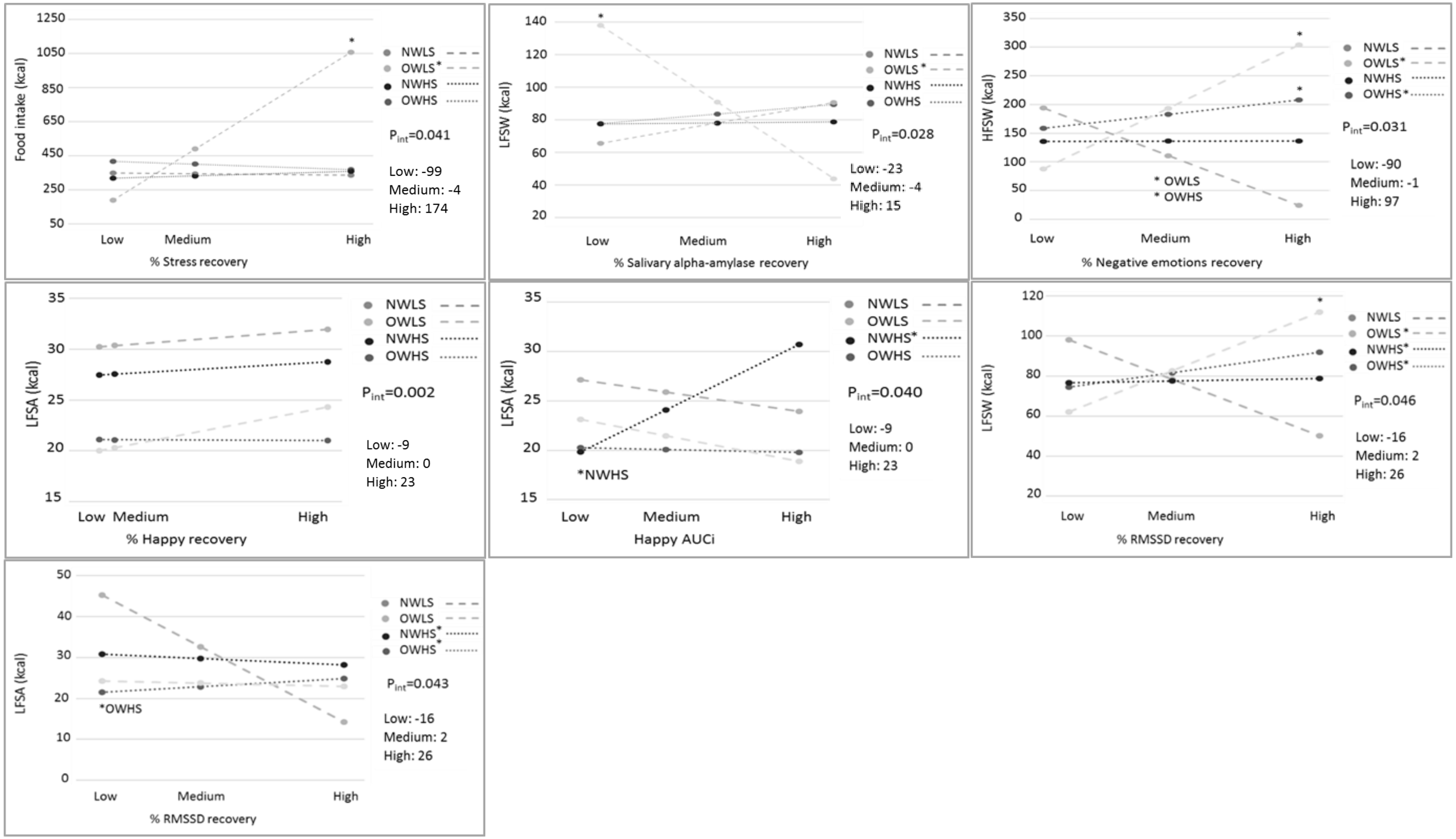

Significant group differences are presented for the stress recovery/AUCi versus food intake relation or for food intake differences at specific stress reactivity levels, with NWLS as reference group. P_{int}= p-value of the group*reactivity interaction; NWLS=Normal Weight Low Stress; OWLS=Overweight Low Stress; NWHS=Normal Weight High Stress; OWHS=Overweight High Stress; HF: high fat; LF: low fat; SW: sweet; SA: savoury.
