# Supplementary material for: Stress Responsiveness and Emotional Eating Depend on Youngsters’ Chronic Stress Level and Overweight
Source: Nutrients. 2021 Oct 19;13(10):3654. doi: 10.3390/nu13103654 (PMC8540677; doi:10.3390/nu13103654)
Supplement: Supplementary file 1 [file nutrients-13-03654-s001.zip › Supplementary Material S1.pdf]

Supplementary Material S1: study strengths and limitations involving the research project

A first strength concerns the methodology and more specific the population sample. Compared to other laboratory studies with time-intensive stress and diet paradigms, the current sample size was large. However, rather low sample sizes were achieved by distributing participants in four subgroups (between 16 and 52). The 18 additional subjects recruited from an obesity medical treatment program were given dietary and psychological aid, but they were not trained on emotion regulation and therefore no influence of therapy was expected on their stress responsiveness and emotional eating behaviour. However, we cannot rule out that they were aware of the stigma on eating in the absence of hunger, which could have affected their self-reports on emotional eating, leading to an underestimation of the real effects. This might be one of the reasons why we could not confirm a direct association of self-reported trait emotional eating with state emotional eating (also highlighted by Bongers and Jansen [51]). As an advantage, it can be reported that the participant sample represented the prevalence of overweight (20.4%), obesity (8.8%) and depressive symptoms (21.2%) when compared to WHO records (i.e., 18-19%, 6-8% and 20%, respectively) [2,3]. A rather high prevalence of moderate stress levels (70.1%) might be due to oversampling of the overweight group [1].

This research is unique in examining stress responsiveness and emotional eating simultaneously in relation to the mix of chronic stress and overweight. A major strength is the mechanistic verification of this stress responsiveness via multiple biomarkers covering the two physiological stress axes and multiple reports, while not only assessing stress reactivity but also stress recovery and total stress responsiveness (i.e. the combination of stress reactivity and recovery). Testing stress reactivity and recovery as moderators towards emotional eating can be regarded as an added value of this work but this large number of analyses did increase the risk of false positive findings and the moderation analyses results should thus be considered exploratory. Since laboratory intervention responses differ from real-life, the current study was designed with great attention to robustness, repeatability and blinding. Indeed, a time-standardized protocol (due to video instructions), a validated stress manipulation test (TSST-C) and an easy-to-believe story involving taste, were set up. As a result, the stress manipulation test proved successful since all direct stress markers commonly described in literature showed significant reactivity (i.e. perceived self-reported stress, sCortisol, sAA, RMSSD). Nevertheless, the TSST-C did not strongly affect mood parameters (happy, sad, scared and mad), which would require a mood manipulation rather than our focus on stress manipulation. A similar design with alternative mood manipulation would indeed be of interest for future research, as it might shed new light on mechanisms involved in emotion-induced eating.

Additional study strengths and limitations involve the food laboratory. To overcome the bias of reluctance to eat in front of others, which is especially seen in children with eating disorders [1], the study was framed towards the participants as testing taste changes instead of studying eating behaviour and participants were left alone without social control when consuming ‘left-overs’. Since multiple studies on emotional eating described selective changes towards an increase in highly palatable snacks (especially high in sweet and fat) instead of “meal” foods

or healthy snacks [17,38,53], this study offered four snack types. Last, the stress exposure successfully increased food wanting (a unique measure of this study), which was reflected in higher snack buffet intake. The concept of 'wanting' and the actual food intake seemed sufficiently distinguished from reported snack 'liking'. Hence, food intake was probably related to stress-induced eating (as intended) rather than hedonic eating. To avoid study bias related to hunger and subsequent eating out of hunger, a preload was given, and hunger was included as confounder.
