# Supplementary material for: Stress Responsiveness and Emotional Eating Depend on Youngsters’ Chronic Stress Level and Overweight
Source: Nutrients. 2021 Oct 19;13(10):3654. doi: 10.3390/nu13103654 (PMC8540677; doi:10.3390/nu13103654)
Supplement: Supplementary file 1 [file nutrients-13-03654-s001.zip › Table S1.pdf]

Table S1. Used food items with corresponding food category and nutrient composition per 100 gram

Food category	Food item	Energy (kcal)	Fat (g)	Fat (kcal)	Sugars (g)	Sugars (kcal)	Sodium (mg)	Total energy per portion (kcal)
Low Fat Sweet	White grapes	71	0.3	2.7	13.3	53.2	1	35.5
	Sweetened cornflakes (Honey Loops)	391	3.5	31.5	27	108	400	195.5
	Gummy bears	343	0.5	4.5	46	184	30	171.5
High Fat Sweet	Brown chocolate	449	17.1	153.9	55	220	58	224.5
	Chocolate chip cookies	510	26	234	31	124	320	255
	M&M’s	501	20.9	187.9	67.8	271.2	200	250.5
Low Fat Savoury	Salted pretzel sticks	347	0.5	4.5	2.3	9.2	2720	173.5
	Puffed Rice Cakes	380	3.8	34.2	2.5	10	75	190
	Crisp bread	372	5.26	47.34	1.6	6.4	750	186
High Fat Savoury	Salted chips	539	35	315	0.5	2	480	269.5
	Roasted & salted peanuts	600	50	450	3.1	12.4	680	300
	TUC salted crackers	486	22	198	7.2	28.8	680	243
