# Supplementary material for: Stress Responsiveness and Emotional Eating Depend on Youngsters’ Chronic Stress Level and Overweight
Source: Nutrients. 2021 Oct 19;13(10):3654. doi: 10.3390/nu13103654 (PMC8540677; doi:10.3390/nu13103654)
Supplement: Supplementary file 1 [file nutrients-13-03654-s001.zip › Table S2.pdf]

Table S2. Descriptive baseline data for continuous variables

**Variable data (n=137)**	**Mean ± SD**	**Median [p25;p75])**	**Minimum**	**Maximum**
Body mass index z-score	0.4±0.4		-2.227	3.539
Perceived stress scale (0-40)	14.2±2.0		2.0	30.0
Depressive symptoms (0-56)	9.0±6.2		0.0	27.0
Hair cortisol (pg/mg)		2.6 [1.9; 3.7]	1.570	51.135
Age	14.2±2.0		6	18
Trait emotional eating (13-65)	27.7±11.8		13.0	65.0
Stress report (1-100)		10 [1; 20]	1	72
Negative emotions report (3-300)		3 [3; 15]	3	139
Happy report (1-100)	66.1±23.7		1	100
HRV RMSSD (ms)		51 [39; 76]	14.2	263.3
HRV stress index	8.3±3.1		2.8	21.6
Salivary cortisol (pg/ml)		311 [115; 693]	8.2	3523.2
Salivary alpha-amylase (nmol/min/ml)	43903±19562		7736.6	124915.7
Hunger (1-100)	16.4±20.2		1	90
Liking (12-1200)	743.9±162.3		227	1081
Wanting (12-1200)	410.0±235.8		12	885

HRV: heart rate variability; RMSSD: Root Mean Square of the Successive Differences
