# Supplementary material for: Stress Responsiveness and Emotional Eating Depend on Youngsters’ Chronic Stress Level and Overweight
Source: Nutrients. 2021 Oct 19;13(10):3654. doi: 10.3390/nu13103654 (PMC8540677; doi:10.3390/nu13103654)
Supplement: Supplementary file 1 [file nutrients-13-03654-s001.zip › Table S3.pdf]

Table S3. Spearman Correlation

Spearman Correlation	Hair cortisol	CDI-2	PSS	BMI z-score	Trait emotional eating	Chronic stress z-score	sAA reactivity	sAA recovery	AUCi sAA	sCort reactivity	sCort recovery	AUCi sCort	Stress reactivity
Hair cortisol	1.000												
CDI-2	0.158	1.000											
PSS	,239**	,623**	1.000										
BMI z-score	0.145	,257**	0.118	1.000									
Trait emotional eating	0.093	,242**	,220**	-0.157	1.000								
Chronic stress z-score	,588**	,787**	,824**	,209*	,239**	1.000							
sAA reactivity				-0.003	-0.144	-0.037	1.000						
sAA recovery				-0.003	-0.072	0.151	-,379**	1.000					
AUCi sAA				0.102	-0.135	0.080	0.156	,517**	1.000				
sCort reactivity				0.121	0.026	0.088	0.136	0.040	,204*	1.000			
sCort recovery				-0.103	-0.056	0.051	-,193*	-0.066	-0.163	-,442**	1.000		
AUCi sCort				-0.022	-0.056	0.014	-0.087	-0.043	-0.068	-,246**	,624**	1.000	
Stress reactivity				-0.039	0.039	0.004	0.003	-0.091	0.030	0.135	-0.049	-0.062	1.000
Stress recovery				0.002	-0.037	0.075	-0.077	-0.043	-0.037	-0.142	,211*	0.173	-,545**
AUCi stress				-0.127	0.061	0.041	-0.112	-0.124	-0.016	-0.162	0.155	0.111	-0.070
Neg. emotions reactivity				0.020	-0.066	0.039	0.149	-0.109	-0.059	0.081	-0.107	0.037	,205*
Neg. Emotions recovery				-0.019	-0.122	-0.115	-0.033	-0.015	-0.027	-0.117	0.131	0.082	-,227**
AUCi Neg. Emotions				-0.018	-0.066	-0.123	0.023	-0.020	-0.046	-0.100	0.014	0.067	-0.067
Happy reactivity				-0.005	-0.070	-,264**	-0.038	-0.052	0.022	-0.126	0.048	-0.040	-0.057
Happy recovery				-,183*	0.085	,244**	0.027	-0.001	-0.083	0.156	-0.003	0.008	0.150
AUCi Happy				-,207*	-0.051	-0.146	-0.054	-0.038	-0.076	-0.043	-0.020	-0.077	0.009
Stressindex reactivity				0.082	-0.007	-0.075	0.056	-0.123	-0.042	-0.003	-0.004	-0.135	-0.031
Stressindex recovery				-0.034	-0.060	0.100	-0.085	0.124	0.002	-0.097	,361**	,287**	-0.038
AUCi Stressindex				0.109	-0.040	-0.001	0.003	-0.107	-0.071	-0.085	0.113	-0.046	-0.051
RMSSD reactivity				-0.126	-0.040	-0.020	-0.058	0.119	0.011	0.008	0.116	0.105	0.043
RMSSD recovery				0.069	0.112	-0.018	0.076	-0.063	0.099	0.183	-,324**	-,187*	0.110
AUCi RMSSD				-0.088	0.044	-0.022	-0.027	,180*	0.144	0.072	-0.009	0.074	0.081
Food intake (g)				0.028	0.001	-0.042	-0.025	-0.070	-0.107	0.024	0.060	0.135	-0.091
Food intake (kcal)				-0.008	0.036	-0.056	-0.042	-0.096	-0.147	-0.002	0.105	0.175	-0.105
Food intake (kcal/g)				-0.162	0.076	-0.075	-0.002	-0.151	-0.166	-0.122	0.158	0.173	-0.151
LFSW (g)				0.054	-0.039	-0.026	-0.013	-0.017	-0.021	0.044	0.059	0.088	-0.062
HFSW (g)				0.167	0.026	0.069	-0.031	-0.123	-0.123	0.011	0.047	0.089	-0.048
LFSA (g)				-0.038	0.004	0.023	-,187*	-0.161	-,314**	-0.085	0.107	0.135	0.036
HFSA (g)				-0.140	0.046	-0.129	-0.046	-0.022	-0.143	-0.023	0.146	,209*	-0.169
LFSW (kcal)				0.023	-0.039	-0.076	-0.018	-0.115	-0.064	-0.041	0.140	0.132	-0.142
HFSW (kcal)				0.164	0.030	0.066	-0.030	-0.121	-0.123	0.008	0.045	0.084	-0.045
LFSA (kcal)				-0.092	0.000	-0.018	-0.166	-,191*	-,334**	-0.087	0.055	0.096	0.071
HFSA (kcal)				-0.139	0.052	-0.130	-0.046	-0.025	-0.138	-0.021	0.155	,211*	-0.168
Total liking				-,225*	0.055	-,184*	0.081	-,174*	-,258**	-0.073	0.030	-0.004	0.039
Wanting bTSST-C				-,342**	,195*	-0.109	0.067	-0.116	-,237**	-0.078	-0.092	-0.048	0.028
Wanting aTSST-C				-,277**	0.091	-0.122	0.029	-0.067	-,206*	-0.159	-0.007	0.079	-0.092
Wanting increase				0.077	-0.099	-0.036	-0.052	0.063	0.033	-,218*	0.130	,182*	-,186*
Hunger increase				-0.086	-0.060	-0.025	-0.068	-0.066	-0.143	-,196*	-0.011	0.080	0.128
Hunger bTSST-C				-,357**	,219*	0.063	-0.003	0.100	-0.133	-0.092	-0.176	-0.105	0.007
Hunger after TSST-C				-,323**	0.085	0.000	-0.031	0.033	-0.155	-0.147	-0.171	-0.043	0.075

Spearman Correlation	Stress recovery	AUCi stress	Neg. emotions reactivity	Neg. Emotions recovery	AUCi Neg. Emotions	Happy reactivity	Happy recovery	AUCi Happy	Stressindex reactivity	Stressindex recovery	AUCi Stressindex	RMSSD reactivity	RMSSD recovery	AUCi RMSSD
Stress recovery	1.000													
AUCi stress	,688**	1.000												
Neg. emotions reactivity	-0.168	-0.160	1.000											
Neg. Emotions recovery	,228**	0.083	-,307**	1.000										
AUCi Neg. Emotions	0.134	0.050	,340**	,627**	1.000									
Happy reactivity	-0.043	0.003	0.041	-0.001	0.035	1.000								
Happy recovery	-0.109	-0.044	0.042	-0.147	-0.139	-,305**	1.000							
AUCi Happy	-0.087	0.028	0.057	-0.130	-0.109	,698**	,279**	1.000						
Stressindex reactivity	0.178	0.149	0.080	0.084	,205*	-0.043	-0.121	-,216*	1.000					
Stressindex recovery	0.080	0.009	0.001	0.105	0.019	0.041	0.156	0.166	-,309**	1.000				
AUCi Stressindex	,214*	0.107	0.123	0.137	,234*	0.000	-0.063	-0.119	,875**	0.083	1.000			
RMSSD reactivity	-0.125	-0.081	-,210*	-0.026	-,205*	0.022	0.099	0.166	-,822**	,200*	-,791**	1.000		
RMSSD recovery	-0.133	-0.092	0.144	-0.125	0.011	-0.064	-0.116	-0.168	,319**	-,818**	0.027	-,307**	1.000	
AUCi RMSSD	-,209*	-0.124	-0.115	-0.090	-,206*	0.045	0.052	0.120	-,691**	-0.117	-,788**	,848**	0.093	1.000
Food intake (g)	0.025	-0.011	0.059	0.068	0.045	0.020	,189*	0.119						
Food intake (kcal)	0.046	-0.005	0.048	0.083	0.070	0.055	0.156	0.121						
Food intake (kcal/g)	0.148	0.043	-0.019	0.041	0.105	0.115	-0.039	0.068						
LFSW (g)	-0.023	-0.046	-0.007	0.040	-0.007	-0.106	0.156	0.008						
HFSW (g)	0.090	-0.015	0.065	0.016	0.016	0.149	0.053	0.167						
LFSA (g)	0.002	0.083	0.145	0.036	0.059	0.015	0.109	0.095						
HFSA (g)	0.006	-0.048	0.083	0.172	0.137	-0.001	,189*	0.065						
LFSW (kcal)	0.078	0.031	-0.004	0.008	0.028	-0.112	0.082	-0.025						
HFSW (kcal)	0.089	-0.017	0.066	0.012	0.015	0.151	0.048	0.167						
LFSA (kcal)	-0.058	0.067	,220*	0.024	0.107	0.010	0.057	0.058						
HFSA (kcal)	0.007	-0.047	0.076	0.171	0.135	0.002	,179*	0.061						
Total liking	-0.012	-0.005	-0.065	0.025	-0.023	0.059	0.067	0.090						
Wanting bTSST-C	-0.078	-0.083	0.067	-0.078	-0.023	0.158	0.126	,234**						
Wanting aTSST-C	0.032	0.005	0.057	-0.035	0.041	0.078	0.165	,177*						
Wanting increase	0.119	0.153	0.016	0.071	0.121	-0.043	0.064	0.012						
Hunger increase	-0.099	-0.021	0.008	0.122	0.086	-0.027	0.085	0.043						
Hunger bTSST-C	0.012	0.078	0.137	-0.072	0.101	0.060	,187*	,210*						
Hunger after TSST-C	-0.050	0.044	0.097	0.012	0.124	0.016	0.163	0.169						

Spearman Correlation	Food intake (g)	Food intake (kcal)	Food intake (kcal/g)	LFSW (g)	HFSW (g)	LFSA (g)	HFSA (g)	LFSW (kcal)	HFSW (kcal)	LFSA (kcal)	HFSA (kcal)	Total liking	Wanting bTSST-C	Wanting aTSST-C	Wanting increase	Hunger increase	Hunger bTSST-C	Hunger after TSST-C
Food intake (g)	1.000																	
Food intake (kcal)	,952**	1.000																
Food intake (kcal/g)	0.088	,361**	1.000															
LFSW (g)	,828**	,656**	-,355**	1.000														
HFSW (g)	,691**	,767**	,393**	,383**	1.000													
LFSA (g)	,411**	,434**	0.132	,228**	,236**	1.000												
HFSA (g)	,744**	,795**	,280**	,468**	,357**	,358**	1.000											
LFSW (kcal)	,783**	,712**	-0.016	,871**	,461**	,274**	,444**	1.000										
HFSW (kcal)	,691**	,766**	,392**	,385**	,999**	,233**	,356**	,460**	1.000									
LFSA (kcal)	,361**	,384**	0.126	,180*	,200*	,996**	,305**	,219*	,198*	1.000								
HFSA (kcal)	,743**	,793**	,279**	,472**	,354**	,352**	,999**	,448**	,352**	,297**	1.000							
Total liking	0.033	0.086	,233**	-0.052	0.108	0.116	0.028	0.015	0.110	0.116	0.028	1.000						
Wanting bTSST-C	,194*	,240**	,233**	0.088	,186*	0.021	,232**	0.130	,190*	0.050	,230**	,529**	1.000					
Wanting aTSST-C	,270**	,322**	,266**	0.138	,215*	0.127	,324**	,205*	,218*	0.165	,319**	,463**	,832**	1.000				
Wanting increase	,228**	,214*	0.007	,214*	0.067	,245**	,221*	,236**	0.067	,294**	,217*	-0.099	-0.106	,377**	1.000			
Hunger increase	0.111	0.165	,207*	-0.029	0.128	,267**	,215*	0.028	0.125	,279**	,206*	0.064	0.133	,258**	,327**	1.000		
Hunger bTSST-C	0.088	0.104	0.080	0.058	0.106	-0.074	0.117	0.061	0.105	-0.015	0.118	0.143	,488**	,404**	-0.051	0.106	1.000	
Hunger after TSST-C	0.154	,200*	,208*	0.046	,181*	0.092	,207*	0.101	,179*	0.155	,204*	0.150	,452**	,486**	,178*	,649**	,745**	1.000

CDI-2: Children´s Depression Inventory; PSS: Perceived Stress Scale; AUCi: Area Under the Curve with respect to increase; sAA:Salivary alpha-amylase; sCort: Salivary cortisol; Neg. Emotions: Negative emotions; RMSSD: Root Mean Square of the Successive Differences; LF: Low Fat; HF: High Fat; SW: Sweet; SA: Savoury
* p<0.05; ** p<0.01

The overall chronic stress score was correlated with BMI z-score (r=0.209). Trait emotional eating seemed significantly reflected in reported food wanting (r=0.195) but not in the snack buffet intake (overall or category specific). Expected correlations were seen between the stress response parameters e.g. reverse correlations between RMSSD and stress index; positive correlations between negative emotions and self-reported stress; positive correlations for sCortisol with self-reported stress and the stress index while negative with RMSSD. Also, food parameters (overall and categories) showed significant mutual positive correlations. Overall liking of the presented snacks was not associated with overall snack buffet intake nor hunger. Hunger was related to consumed calories. BMI was negatively associated with wanting and hunger (before or after stress induction) and with overall liking, while chronic stress was only negatively associated with liking. As expected, the increase in wanting was associated with higher overall kcal intake of the snack buffet and with LFSW, LFSA, HFSA snack categories but not with total liking. As expected, no significant correlations were found between hair cortisol and salivary cortisol reactivity.
