# Supplementary material for: Stress Responsiveness and Emotional Eating Depend on Youngsters’ Chronic Stress Level and Overweight
Source: Nutrients. 2021 Oct 19;13(10):3654. doi: 10.3390/nu13103654 (PMC8540677; doi:10.3390/nu13103654)
Supplement: Supplementary file 1 [file nutrients-13-03654-s001.zip › Table S4.pdf]

Table S4. BMI and chronic stress level as predictor of stress response and food parameters after Trier Social Stress Test induction.

		Separate model		Model with both predictors	
		**BMI z-score**	**Chronic stress z-score**	**BMI z-score**	**Chronic stress z-score**
**% Salivary alpha-amylase reactivity**	β	-0.044	0.035	-0.047	0.041
	p	0.638	0.713	0.616	0.671
**% Salivary alpha-amylase recovery**	β	0.034	0.043	0.032	0.039
	p	0.715	0.657	0.730	0.691
**AUCi Salivary alpha-amylase**	β	0.146	-0.006	0.151	-0.025
	p	0.113	0.947	0.107	0.794
**% Salivary cortisol reactivity**	β	0.171	0.031	0.166	0.014
	p	0.086	0.770	0.100	0.891
**% Salivary cortisol recovery**	β	0.099	0.072	0.096	0.065
	p	0.325	0.491	0.344	0.535
**AUCi Salivary cortisol**	β	-0.021	-0.051	-0.015	-0.049
	p	0.821	0.607	0.878	0.626
**% Stress increase**	β	0.079	-0.005	0.083	-0.015
	p	0.385	0.962	0.153	0.897
**% Stress recovery**	β	-0.031	0.151	-0.047	0.157
	p	0.737	0.116	0.617	0.107
**AUCi Stress**	β	-0.169	0.015	-0.169	0.036
	p	0.062	0.875	0.065	0.706
**% Negative emotions reactivity**	β	0.077	0.072	0.072	0.064
	p	0.403	0.452	0.442	0.513
**% Negative emotions recovery**	β	-0.004	0.126	-0.017	0.128
	p	0.965	0.187	0.855	0.185
**AUCi Negative emotions**	β	-0.015	-0.027	-0.012	-0.026
	p	0.874	0.779	0.902	0.792
**% Happy reactivity**	β	0.087	-0.037	0.091	-0.048
	p	0.343	0.698	0.324	0.616
**% Happy recovery**	β	0.114	0.117	0.101	0.104
	p	0.218	0.224	0.277	0.280
**AUCi happy**	β	-0.033	-0.030	-0.026	-0.027
	p	0.726	0.759	0.783	0.786
**% Stress index reactivity**	β	0.020	0.116	0.017	0.115
	p	0.835	0.233	0.863	0.241
**% Stress index recovery**	β	-0.041	0.068	-0.036	0.071
	p	0.662	0.478	0.708	0.465
**AUCi Stress index**	β	0.040	0.100	0.056	0.096
	p	0.661	0.285	0.545	0.306
**% RMSSD reactivity**	β	-0.052	0.008	-0.052	0.011
	p	0.585	0.938	0.590	0.911
**% RMSSD recovery**	β	0.024	0.001	0.023	-0.001
	p	0.801	0.992	0.814	0.996
**AUCi RMSSD**	β	0.048	-0.104	0.053	-0.107
	p	0.600	0.267	0.563	0.254
**Increase in hunger**	β	-0.035	0.055	-0.041	0.060
	p	0.727	0.574	0.684	0.543
**Increase in wanting**	β	0.056	0.004	0.056	-0.003
	p	0.566	0.969	0.571	0.972
**Trait emotional eating**	β	-0.105	0.213	-0.136	0.164
	p	0.288	**0.024**	0.230	**0.016**
**Food intake** (kcal)	β	0.060	0.024	0.051	0.018
	p	0.536	0.794	0.605	0.849
**Energy density intake** (kcal/g)	β	-0.105	0.102	-0.123	0.118
	p	0.270	0.267	0.199	0.204

**HFSW intake** (kcal)	β	0.247	0.165	0.223	0.137
	p	**0.011**	0.080	**0.022**	0.141
**LFSW intake** (kcal)	β	0.078	-0.016	0.072	-0.025
	p	0.428	0.865	0.466	0.792
**HFSA intake** (kcal)	β	-0.125	-0.101	-0.114	-0.081
	p	0.211	0.314	0.266	0.426
**LFSA intake** (kcal)	β	-0.130	0.001	-0.134	0.020
	p	0.215	0.989	0.210	0.849

Results are given for the separate models including chronic stress z-score or BMI z-score, and for the combined model. Β: standardized regression coefficients from linear regression adjusted for age, sex and parental education. Wanting and snack buffet intake analyses were additionally adjusted for hunger at study start and liking of presented snacks. AUCi: area under the curve with respect to increase; RMSSD: Root Mean Square of the Successive Differences HF: high fat; LF: low fat; SW: sweet; SA: savoury.
