# Supplementary material for: Stress Responsiveness and Emotional Eating Depend on Youngsters’ Chronic Stress Level and Overweight
Source: Nutrients. 2021 Oct 19;13(10):3654. doi: 10.3390/nu13103654 (PMC8540677; doi:10.3390/nu13103654)
Supplement: Supplementary file 1 [file nutrients-13-03654-s001.zip › Table S5.pdf]

Table S5. Moderating effects of stress measures: reactivity, recovery or AUCi on associations between chronic stress weight groups and food intake

		Moderator				
		% stress increase				
**Food lab responses**		**b_{int}**	**SE_{int}**	**p_{int}**	**ΔR^2_{int}**	**R^2**
**Food intake (kcal)**	Overweight & low stress n=16	0.0028	0.0527	0.9582		
	Normal weight & high stress n=45	0.0710	0.0316	0.0264		
	Overweight & high stress n=23	0.0313	0.0330	0.3450		
	Total model			0.1564	0.0384	0.1762
**Energy density intake (kcal/g)**	Overweight & low stress n=16	0.0000	0.0001	0.7829		
	Normal weight & high stress n=45	0.0000	0.0001	0.8833		
	Overweight & high stress n=23	0.0001	0.0001	0.4686		
	Total model			0.8696	0.8696	0.1640
**HFSW intake (kcal)**	Overweight & low stress n=16	0.0015	0.0247	0.9512		
	Normal weight & high stress n=45	0.0276	0.0148	0.0649		
	Overweight & high stress n=23	0.0071	0.0155	0.6477		
	Total model			0.3118	0.0255	0.1940
**LFSW intake (kcal)**	Overweight & low stress n=16	0.0001	0.0138	0.9916		
	Normal weight & high stress n=45	0.0224	0.0082	**0.0075**		
	Overweight & high stress n=23	0.0124	0.0086	0.1541		
	Total model			**0.0481**	0.0587	0.1785
**HFSA intake (kcal)**	Overweight & low stress n=16	0.0089	0.0341	0.7944		
	Normal weight & high stress n=45	0.0181	0.0162	0.2654		
	Overweight & high stress n=23	0.0120	0.0169	0.4787		
	Total model			0.7109	0.0113	0.1076
**LFSA intake (kcal)**	Overweight & low stress n=16	0.0064	0.0086	0.4573		
	Normal weight & high stress n=45	0.0011	0.0041	0.7799		
	Overweight & high stress n=23	-0.0001	0.0043	0.9856		
	Total model			0.8935	0.0055	0.0391
		% stress recovery				
**Food lab responses**		**b_{int}**	**SE_{int}**	**p_{int}**	**ΔR^2_{int}**	**R^2**
**Food intake (kcal)**	Overweight & low stress n=16	3.2217	1.3234	**0.0165**		
	Normal weight & high stress n=45	0.1953	0.5514	0.7238		

	Overweight & high stress n=23	-0.1366	0.5772	0.8134		
	Total model			**0.0411**	0.0596	0.2028
**Energy density intake (kcal/g**	Overweight & low stress n=16	0.0040	0.0038	0.2890		
	Normal weight & high stress n=45	-0.0008	0.0016	0.6080		
	Overweight & high stress n=23	-0.0011	0.0016	0.5046		
	Total model			0.4831	0.0175	0.1918
**HFSW intake (kcal)**	Overweight & low stress n=16	0.1520	0.6309	0.0705		
	Normal weight & high stress n=45	0.0544	0.2629	0.8365		
	Overweight & high stress n=23	-0.0514	0.2752	0.8521		
	Total model			0.2063	0.0328	0.1942
**LFSW intake (kcal)**	Overweight & low stress n=16	0.7198	0.3516	0.0429		
	Normal weight & high stress n=45	-0.0830	0.1465	0.5722		
	Overweight & high stress n=23	-0.1512	0.1534	0.3262		
	Total model			0.0512	0.0576	0.1786
**HFSA intake (kcal)**	Overweight & low stress n=16	1.4372	0.7296	0.0514		
	Normal weight & high stress n=45	0.4330	0.2823	0.1279		
	Overweight & high stress n=23	0.2706	0.2953	0.3615		
	Total model			0.1175	0.0476	0.1368
**LFSA intake (kcal)**	Overweight & low stress n=16	-0.1358	0.1818	0.4565		
	Normal weight & high stress n=45	-0.1838	0.0715	0.0116		
	Overweight & high stress n=23	-0.1872	0.0748	0.0138		
	Total model			0.0843	0.0584	0.0923
		**AUCi stress**				
**Food lab responses**		**b_{int}**	**SE_{int}**	**p_{int}**	**ΔR^2_{int}**	**R^2**
**Food intake (kcal)**	Overweight & low stress n=16	0.2637	0.3100	0.3967		
	Normal weight & high stress n=45	-0.0293	0.2684	0.9132		
	Overweight & high stress n=23	0.0366	0.3242	0.9104		
	Total model			0.7652	0.0086	0.1482
**Energy density intake (kcal/g)**	Overweight & low stress n=16	-0.0001	0.0008	0.9522		
	Normal weight & high stress n=45	-0.0010	0.0007	0.1852		
	Overweight & high stress n=23	0.0007	0.0009	0.4265		
	Total model			0.1585	0.0370	0.2013

**HFSW intake (kcal)**	Overweight & low stress n=16	0.1303	0.1439	0.3670		
	Normal weight & high stress n=45	0.0108	0.1245	0.9311		
	Overweight & high stress n=23	0.2306	0.1505	0.1282		
	Total model			0.3112	0.0259	0.1841
**LFSW intake (kcal)**	Overweight & low stress n=16	0.0397	0.0817	0.6280		
	Normal weight & high stress n=45	-0.0392	0.0707	0.5800		
	Overweight & high stress n=23	-0.0721	0.0854	0.4001		
	Total model			0.6051	0.0140	0.1373
**HFSA intake (kcal)**	Overweight & low stress n=16	0.1891	0.3048	0.5363		
	Normal weight & high stress n=45	0.0433	0.1369	0.7525		
	Overweight & high stress n=23	-0.0779	0.1647	0.6374		
	Total model			0.7763	0.0092	0.0920
**LFSA intake (kcal)**	Overweight & low stress n=16	-0.0751	0.0771	0.3324		
	Normal weight & high stress n=45	-0.0230	0.0338	0.4974		
	Overweight & high stress n=23	-0.0430	0.0408	0.2944		
	Total model			0.6478	0.0147	0.0570
		**% Salivary alpha-amylase reactivity**				
**Food lab responses**		**b_{int}**	**SE_{int}**	**p_{int}**	**ΔR^2_{int}**	**R^2**
**Food intake (kcal)**	Overweight & low stress n=16	-1.4383	4.4548	0.7474		
	Normal weight & high stress n=45	0.3573	2.7556	0.8971		
	Overweight & high stress n=23	-2.6382	3.9225	0.5026		
	Total model			0.8792	0.0051	0.1426
**Energy density intake (kcal/g)**	Overweight & low stress n=16	-0.0135	0.0123	0.2772		
	Normal weight & high stress n=45	0.0023	0.0076	0.7635		
	Overweight & high stress n=23	0.0003	0.0109	0.9801		
	Total model			0.6492	0.0119	0.1749
**HFSW intake (kcal)**	Overweight & low stress n=16	-1.8939	2.0833	0.3652		
	Normal weight & high stress n=45	-0.4146	1.2886	0.7482		
	Overweight & high stress n=23	-0.3542	1.8344	0.8472		
	Total model			0.8418	0.0061	0.1660
**LFSW intake (kcal)**	Overweight & low stress n=16	0.8592	1.1738	0.4657		
	Normal weight & high stress n=45	0.3643	0.7261	0.6168		

	Overweight & high stress n=23	-0.4633	1.0335	0.6548		
	Total model			0.7469	0.0093	0.1311
**HFSA intake (kcal)**	Overweight & low stress n=16	-1.0320	2.3793	0.6654		
	Normal weight & high stress n=45	-0.2326	1.4368	0.8717		
	Overweight & high stress n=23	-2.0104	1.9872	0.3139		
	Total model			0.7741	0.0093	0.0917
**LFSA intake (kcal)**	Overweight & low stress n=16	-0.0196	0.6490	0.9759		
	Normal weight & high stress n=45	0.4829	0.3591	0.1816		
	Overweight & high stress n=23	0.1617	0.4916	0.7429		
	Total model			0.5852	0.0173	0.0577
		**% Salivary alpha-amylase recovery**				
**Food lab responses**		b_{int}	SE_{int}	p_{int}	ΔR^2_{int}	R^2
**Food intake (kcal)**	Overweight & low stress n=16	-5.9788	4.0968	0.1472		
	Normal weight & high stress n=45	-1.4445	2.0744	0.4876		
	Overweight & high stress n=23	0.9354	4.1555	0.8223		
	Total model			0.4771	0.0186	0.1562
**Energy density intake (kcal/g)**	Overweight & low stress n=16	0.0123	0.0114	0.2834		
	Normal weight & high stress n=45	0.0003	0.0058	0.9638		
	Overweight & high stress n=23	-0.0053	0.0115	0.6450		
	Total model			0.6809	0.0108	0.1845
**HFSW intake (kcal)**	Overweight & low stress n=16	0.0085	1.9135	0.9965		
	Normal weight & high stress n=45	0.2883	0.9689	0.7666		
	Overweight & high stress n=23	3.3705	1.9409	0.8520		
	Total model			0.3877	0.0219	0.1813
**LFSW intake (kcal)**	Overweight & low stress n=16	-3.1766	1.0505	**0.0031**		
	Normal weight & high stress n=45	-0.6317	0.5319	0.2375		
	Overweight & high stress n=23	-0.3634	1.0656	0.7337		
	Total model			**0.0277**	**0.0672**	0.1901
**HFSA intake (kcal)**	Overweight & low stress n=16	-2.7821	2.2760	0.2242		
	Normal weight & high stress n=45	-0.9658	1.0560	0.3624		
	Overweight & high stress n=23	-2.0920	2.1068	0.3229		
	Total model			0.4702	0.0209	0.1032

**LFSA intake (kcal)**	Overweight & low stress n=16	0.1481	0.5279	0.7796		
	Normal weight & high stress n=45	-0.1552	0.2719	0.5694		
	Overweight & high stress n=23	-0.0764	0.5335	0.8865		
	Total model			0.9176	0.0046	0.0416
		**AUCi Salivary alpha-amylase**				
**Food lab responses**		**b_{int}**	**SE_{int}**	**p_{int}**	**ΔR^2_{int}**	**R^2**
**Food intake (kcal)**	Overweight & low stress n=16	-0.0025	0.0015	0.1003		
	Normal weight & high stress n=45	0.0001	0.0004	0.7928		
	Overweight & high stress n=23	0.0001	0.0004	0.8677		
	Total model			0.4046	0.0217	0.1595
**Energy density intake (kcal/g)**	Overweight & low stress n=16	0.0000	0.0000	0.5723		
	Normal weight & high stress n=45	0.0000	0.0000	0.4361		
	Overweight & high stress n=23	0.0000	0.0000	0.9885		
	Total model			0.7905	0.0076	0.1662
**HFSW intake (kcal)**	Overweight & low stress n=16	-0.0007	0.0007	0.3489		
	Normal weight & high stress n=45	0.0001	0.0002	0.7461		
	Overweight & high stress n=23	0.0002	0.0002	0.3097		
	Total model			0.5471	0.0154	0.1764
**LFSW intake (kcal)**	Overweight & low stress n=16	-0.0008	0.0004	0.0497		
	Normal weight & high stress n=45	0.0001	0.0001	0.1149		
	Overweight & high stress n=23	0.0000	0.0001	0.8390		
	Total model			0.0780	0.0508	0.1712
**HFSA intake (kcal)**	Overweight & low stress n=16	-0.0009	0.0009	0.3170		
	Normal weight & high stress n=45	-0.0001	0.0002	0.5500		
	Overweight & high stress n=23	-0.0001	0.0002	0.4716		
	Total model			0.6697	0.0129	0.0994
**LFSA intake (kcal)**	Overweight & low stress n=16	0.0000	0.0002	0.9576		
	Normal weight & high stress n=45	0.0000	0.0000	0.7896		
	Overweight & high stress n=23	0.0000	0.0001	0.9762		
	Total model			0.9941	0.0267	0.0603
		**% Salivary cortisol reactivity**				
**Food lab responses**		**b_{int}**	**SE_{int}**	**p_{int}**	**ΔR^2_{int}**	**R^2**

**Food intake (kcal)**	Overweight & low stress n=16	-0.1208	0.2164	0.5779		
	Normal weight & high stress n=45	0.0003	0.0938	0.9972		
	Overweight & high stress n=23	-0.0256	0.0670	0.7029		
	Total model			0.9054	0.0049	0.1746
**Energy density intake (kcal/g)**	Overweight & low stress n=16	-0.0008	0.0006	0.1707		
	Normal weight & high stress n=45	-0.0003	0.0003	0.3231		
	Overweight & high stress n=23	-0.0001	0.0002	0.4768		
	Total model			0.4783	0.0202	0.2320
**HFSW intake (kcal)**	Overweight & low stress n=16	-0.0451	0.1057	0.6706		
	Normal weight & high stress n=45	0.0000	0.0458	0.9999		
	Overweight & high stress n=23	-0.0093	0.0327	0.7768		
	Total model			0.9570	0.0027	0.1973
**LFSW intake (kcal)**	Overweight & low stress n=16	-0.0219	0.0589	0.7112		
	Normal weight & high stress n=45	0.0226	0.0255	0.3783		
	Overweight & high stress n=23	-0.0103	0.0182	0.5742		
	Total model			0.2323	0.0375	0.1829
**HFSA intake (kcal)**	Overweight & low stress n=16	-0.0742	0.1014	0.4659		
	Normal weight & high stress n=45	-0.028	0.044	0.5260		
	Overweight & high stress n=23	-0.0165	0.0314	0.5995		
	Total model			0.8529	0.0077	0.1097
**LFSA intake (kcal)**	Overweight & low stress n=16	0.0106	0.0333	0.7503		
	Normal weight & high stress n=45	0.0146	0.0183	0.4266		
	Overweight & high stress n=23	0.0151	0.0159	0.3456		
	Total model			0.8178	0.0102	0.0491
		**% Salivary cortisol recovery**				
**Food lab responses**		**b_{int}**	**SE_{int}**	**p_{int}**	**ΔR^2_{int}**	**R^2**
**Food intake (kcal)**	Overweight & low stress n=16	5.3823	4.6385	0.2494		
	Normal weight & high stress n=45	0.2796	0.8081	0.7303		
	Overweight & high stress n=23	0.1959	0.8729	0.8230		
	Total model			0.7072	0.0135	0.2277
**Energy density intake (kcal/g)**	Overweight & low stress n=16	0.0113	0.0126	0.3701		
	Normal weight & high stress n=45	-0.0036	0.0022	0.1004		

	Overweight & high stress n=23	-0.0012	0.0024	0.6227		
	Total model			0.2448	0.0414	0.2191
**HFSW intake (kcal)**	Overweight & low stress n=16	1.2981	2.3810	0.5871		
	Normal weight & high stress n=45	0.1484	0.4148	0.7214		
	Overweight & high stress n=23	-0.0832	0.4481	0.8532		
	Total model			0.8865	0.0064	0.2021
**LFSW intake (kcal)**	Overweight & low stress n=16	1.4209	1.2954	0.2760		
	Normal weight & high stress n=45	0.0727	0.2257	0.7482		
	Overweight & high stress n=23	-0.0107	0.2438	0.9651		
	Total model			0.7179	0.0316	0.1915
**HFSA intake (kcal)**	Overweight & low stress n=16	2.6913	2.2808	0.2417		
	Normal weight & high stress n=45	0.1772	0.3828	0.6447		
	Overweight & high stress n=23	0.2901	0.0694	0.7175		
	Total model			0.6223	0.0192	0.1795
**LFSA intake (kcal)**	Overweight & low stress n=16	0.4137	0.6774	0.5433		
	Normal weight & high stress n=45	0.0137	0.1214	0.9602		
	Overweight & high stress n=23	0.0160	0.1273	0.9002		
	Total model			0.9444	0.0048	0.0949
		**AUCi Salivary cortisol**				
**Food lab responses**		b_{int}	SE_{int}	p_{int}	ΔR^2_{int}	R^2
**Food intake (kcal)**	Overweight & low stress n=16	0.0069	0.0069	0.3175		
	Normal weight & high stress n=45	0.0008	0.0010	0.4541		
	Overweight & high stress n=23	0.0015	0.0039	0.7038		
	Total model			0.6342	0.0131	0.1716
**Energy density intake (kcal/g)**	Overweight & low stress n=16	0.0000	0.0000	0.6003		
	Normal weight & high stress n=45	0.0000	0.0000	0.7362		
	Overweight & high stress n=23	0.0000	0.0000	0.8392		
	Total model			0.9328	0.0032	0.2015
**HFSW intake (kcal)**	Overweight & low stress n=16	-0.0001	0.0032	0.9841		
	Normal weight & high stress n=45	0.0007	0.0005	0.1519		
	Overweight & high stress n=23	-0.0013	0.0018	0.4905		
	Total model			0.4711	0.0189	0.1891

**LFSW intake (kcal)**	Overweight & low stress n=16	0.0013	0.0018	0.4858		
	Normal weight & high stress n=45	0	0.0003	0.9484		
	Overweight & high stress n=23	0.0008	0.001	0.4542		
	Total model			0.7883	0.0081	0.1665
**HFSA intake (kcal)**	Overweight & low stress n=16	0.0077	0.0036	0.0342		
	Normal weight & high stress n=45	-0.0001	0.0005	0.8474		
	Overweight & high stress n=23	0.002	0.0019	0.3055		
	Total model			0.1341	0.0473	0.1373
**LFSA intake (kcal)**	Overweight & low stress n=16	-0.0005	0.0009	0.5784		
	Normal weight & high stress n=45	0.0002	0.0001	0.1586		
	Overweight & high stress n=23	0.0000	0.0005	0.9956		
	Total model			0.5104	0.0215	0.0662
		**% neg. emotions reactivity**				
**Food lab responses**		**b_{int}**	**SE_{int}**	**p_{int}**	**ΔR^2_{int}**	**R^2**
**Food intake (kcal)**	Overweight & low stress n=16	-0.6432	0.5546	0.2486		
	Normal weight & high stress n=45	0.0044	0.2554	0.9863		
	Overweight & high stress n=23	-0.0623	0.2469	0.8014		
	Total model			0.7034	0.0104	0.163
**Energy density intake (kcal/g)**	Overweight & low stress n=16	-0.0008	0.0016	0.6058		
	Normal weight & high stress n=45	-0.0004	0.0007	0.6156		
	Overweight & high stress n=23	-0.0006	0.0007	0.3776		
	Total model			0.8177	0.0068	0.1656
**HFSW intake (kcal)**	Overweight & low stress n=16	-0.2229	0.2606	0.3943		
	Normal weight & high stress n=45	0.0618	0.12	0.6073		
	Overweight & high stress n=23	0.0332	0.116	0.7753		
	Total model			0.7494	0.0088	0.1777
**LFSW intake (kcal)**	Overweight & low stress n=16	-0.1533	0.1469	0.2990		
	Normal weight & high stress n=45	-0.0374	0.0677	0.5819		
	Overweight & high stress n=23	-0.0551	0.0654	0.4013		
	Total model			0.6713	0.0117	0.1425
**HFSA intake (kcal)**	Overweight & low stress n=16	1.1147	3.2404	0.7315		
	Normal weight & high stress n=45	-0.0655	0.1309	0.6177		

	Overweight & high stress n=23	-0.0616	0.1263	0.6266		
	Total model			0.9263	0.0039	0.0948
**LFSA intake (kcal)**	Overweight & low stress n=16	-0.151	0.1245	0.2277		
	Normal weight & high stress n=45	-0.1111	0.1101	0.3151		
	Overweight & high stress n=23	-0.1277	0.1095	0.2465		
	Total model			0.6336	0.0153	0.0551
		**% neg. emotions recovery**				
**Food lab responses**		**b_{int}**	**SE_{int}**	**p_{int}**	**ΔR^2_{int}**	**R^2**
**Food intake (kcal)**	Overweight & low stress n=16	3.3269	1.7504	0.0599		
	Normal weight & high stress n=45	0.8209	1.0511	0.4364		
	Overweight & high stress n=23	0.693	1.0727	0.5196		
	Total model			0.2778	0.0283	0.1741
**Energy density intake (kcal/g)**	Overweight & low stress n=16	0.0109	0.0048	0.0247		
	Normal weight & high stress n=45	0.0067	0.0029	0.0211		
	Overweight & high stress n=23	0.0077	0.0029	0.0102		
	Total model			0.0516	0.0541	0.2277
**HFSW intake (kcal)**	Overweight & low stress n=16	2.0542	0.8031	**0.0118**		
	Normal weight & high stress n=45	0.9069	0.4822	0.0626		
	Overweight & high stress n=23	1.1687	0.4922	**0.0192**		
	Total model			**0.0313**	0.0621	0.2268
**LFSW intake (kcal)**	Overweight & low stress n=16	0.9621	0.4625	0.0397		
	Normal weight & high stress n=45	0.1841	0.2777	0.5088		
	Overweight & high stress n=23	0.1324	0.2834	0.6414		
	Total model			0.1708	0.0377	0.1584
**HFSA intake (kcal)**	Overweight & low stress n=16	-0.1302	1.0494	0.9015		
	Normal weight & high stress n=45	-0.3008	0.5335	0.5740		
	Overweight & high stress n=23	-0.6149	0.5443	0.2611		
	Total model			0.3705	0.0254	0.0254
**LFSA intake (kcal)**	Overweight & low stress n=16	0.0624	0.2458	0.8002		
	Normal weight & high stress n=45	-0.0028	0.136	0.9836		
	Overweight & high stress n=23	-0.0254	0.1386	0.8549		
	Total model			0.9551	0.0029	0.0379

		AUCi neg. emotions				
**Food lab responses**		**b_{int}**	**SE_{int}**	**p_{int}**	**ΔR^2_{int}**	**R^2**
**Food intake (kcal)**	Overweight & low stress n=16	0.5713	0.4825	0.2389		
	Normal weight & high stress n=45	0.0429	0.4034	0.9156		
	Overweight & high stress n=23	0.1428	0.5068	0.7787		
	Total model			0.5361	0.0162	0.1588
**Energy density intake (kcal/g)**	Overweight & low stress n=16	0.0015	0.0013	0.2549		
	Normal weight & high stress n=45	0.0000	0.0011	0.9908		
	Overweight & high stress n=23	0.0006	0.0014	0.6524		
	Total model			0.5065	0.0169	0.1795
**HFSW intake (kcal)**	Overweight & low stress n=16	0.4430	0.2239	0.0503		
	Normal weight & high stress n=45	0.1053	0.1872	0.5751		
	Overweight & high stress n=23	0.2113	0.2352	0.3710		
	Total model			0.1818	0.0350	0.1942
**LFSW intake (kcal)**	Overweight & low stress n=16	0.1878	0.1275	0.1436		
	Normal weight & high stress n=45	0.0445	0.1066	0.6771		
	Overweight & high stress n=23	0.0368	0.1339	0.7840		
	Total model			0.4302	0.0209	0.1425
**HFSA intake (kcal)**	Overweight & low stress n=16	-0.0976	0.2495	0.6963		
	Normal weight & high stress n=45	0.0583	0.2174	0.7891		
	Overweight & high stress n=23	-0.0583	0.2572	0.8211		
	Total model			0.8864	0.0053	0.1043
**LFSA intake (kcal)**	Overweight & low stress n=16	-0.0433	0.0632	0.4946		
	Normal weight & high stress n=45	-0.0484	0.0522	0.3557		
	Overweight & high stress n=23	-0.0657	0.0649	0.3134		
	Total model			0.7614	0.0104	0.0517
		**% Happy reactivity**				
**Food lab responses**		**b_{int}**	**SE_{int}**	**p_{int}**	**ΔR^2_{int}**	**R^2**
**Food intake (kcal)**	Overweight & low stress n=16	-3.8715	2.6106	0.1408		
	Normal weight & high stress n=45	0.4137	0.3790	0.2774		
	Overweight & high stress n=23	0.1035	0.0464	**0.0275**		
	Total model			**0.0456**	0.0575	0.2072

**Energy density intake (kcal/g)**	Overweight & low stress n=16	0.0075	0.0074	0.3151		
	Normal weight & high stress n=45	0.0013	0.0011	0.2295		
	Overweight & high stress n=23	-0.0001	0.0001	0.5058		
	Total model			0.3965	0.0213	0.1910
**HFSW intake (kcal)**	Overweight & low stress n=16	-1.2204	1.2157	0.3176		
	Normal weight & high stress n=45	0.1577	0.1765	0.3735		
	Overweight & high stress n=23	0.0619	0.0216	**0.0049**		
	Total model			**0.0228**	0.0666	0.2353
**LFSW intake (kcal)**	Overweight & low stress n=16	-1.1506	0.6944	0.1003		
	Normal weight & high stress n=45	-0.0474	0.1008	0.6390		
	Overweight & high stress n=23	0.0285	0.0123	**0.0228**		
	Total model			**0.0426**	0.0606	0.1812
**HFSA intake (kcal)**	Overweight & low stress n=16	-1.3522	1.3535	0.3200		
	Normal weight & high stress n=45	0.1638	0.1967	0.4068		
	Overweight & high stress n=23	0.0133	0.0240	0.5809		
	Total model			0.5796	0.0159	0.1245
**LFSA intake (kcal)**	Overweight & low stress n=16	-0.2062	0.3371	0.5420		
	Normal weight & high stress n=45	0.1316	0.0481	0.0073		
	Overweight & high stress n=23	-0.0003	0.0059	0.9594		
	Total model			0.0545	0.0664	0.1048
		**% Happy recovery**				
**Food lab responses**		**b_{int}**	**SE_{int}**	**p_{int}**	**ΔR^2_{int}**	**R^2**
**Food intake (kcal)**	Overweight & low stress n=16	1.4273	2.0866	0.4953		
	Normal weight & high stress n=45	-1.5672	0.9686	0.1084		
	Overweight & high stress n=23	-1.6305	0.9683	0.0950		
	Total model			0.1389	0.0402	0.1823
**Energy density intake (kcal/g)**	Overweight & low stress n=16	-0.0038	0.0059	0.5278		
	Normal weight & high stress n=45	-0.0025	0.0028	0.3688		
	Overweight & high stress n=23	-0.0024	0.0028	0.3878		
	Total model			0.8123	0.0007	0.1660
**HFSW intake (kcal)**	Overweight & low stress n=16	0.785	0.9959	0.4322		
	Normal weight & high stress n=45	-0.197	0.4623	0.6708		

	Overweight & high stress n=23	-0.1984	0.4622	0.6686		
	Total model			0.7107	0.0100	0.1715
**LFSW intake (kcal)**	Overweight & low stress n=16	0.5802	0.5553	0.2983		
	Normal weight & high stress n=45	-0.2951	0.2578	0.2548		
	Overweight & high stress n=23	-0.2927	0.2577	0.2585		
	Total model			0.2353	0.0320	0.1544
**HFSA intake (kcal)**	Overweight & low stress n=16	0.1018	1.1017	0.9265		
	Normal weight & high stress n=45	-1.0389	0.4920	0.0370		
	Overweight & high stress n=23	-1.0403	0.4919	0.0367		
	Total model			0.1298	0.0459	0.1328
	Total model			**0.0013**	0.1258	0.1606
**LFSA intake (kcal)**	Overweight & low stress n=16	0.1036	0.5383	0.8477		
	Normal weight & high stress n=45	-0.0173	0.1190	0.8850		
	Overweight & high stress n=23	-0.0705	0.1191	0.5551		
	Total model			**0.0021**	0.1246	0.1605
		**AUCi Happy**				
**Food lab responses**		**b_{int}**	**SE_{int}**	**p_{int}**	**ΔR^2_{int}**	**R^2**
**Food intake (kcal)**	Overweight & low stress n=16	0.1378	0.4103	0.7376		
	Normal weight & high stress n=45	0.2006	0.2359	0.3969		
	Overweight & high stress n=23	0.5322	0.3092	0.0879		
	Total model			0.3615	0.0234	0.1752
**Energy density intake (kcal/g)**	Overweight & low stress n=16	0.0005	0.0012	0.6410		
	Normal weight & high stress n=45	-0.0004	0.0007	0.5568		
	Overweight & high stress n=23	0.0000	0.0009	0.9784		
	Total model			0.8820	0.0048	0.1652
**HFSW intake (kcal)**	Overweight & low stress n=16	-0.0017	0.1895	0.9929		
	Normal weight & high stress n=45	0.0073	0.1090	0.9465		
	Overweight & high stress n=23	0.3098	0.1428	0.0321		
	Total model			0.1867	0.0335	0.2173

**LFSW intake (kcal)**	Overweight & low stress n=16	0.0552	0.1099	0.6164		
	Normal weight & high stress n=45	0.0091	0.0632	0.8857		
	Overweight & high stress n=23	0.1195	0.0828	0.1517		
	Total model			0.5248	0.0170	0.1369
**HFSA intake (kcal)**	Overweight & low stress n=16	0.0605	0.2207	0.7846		
	Normal weight & high stress n=45	0.054	0.1415	0.7038		
	Overweight & high stress n=23	0.0843	0.1596	0.5984		
	Total model			0.9393	0.0034	0.0916
**LFSA intake (kcal)**	Overweight & low stress n=16	-0.0082	0.0729	0.9101		
	Normal weight & high stress n=45	0.1101	0.0379	**0.0045**		
	Overweight & high stress n=23	0.0211	0.0384	0.5840		
	Total model			**0.0401**	0.0723	0.1092
		**% Stress index reactivity**				
**Food lab responses**		**b_{int}**	**SE_{int}**	**p_{int}**	**ΔR^2_{int}**	**R^2**
**Food intake (kcal)**	Overweight & low stress n=16	1.3611	2.9998	0.6509		
	Normal weight & high stress n=45	1.1416	1.8815	0.5453		
	Overweight & high stress n=23	2.9747	3.1022	0.3398		
	Total model			0.8116	0.0078	0.1496
**Energy density intake (kcal/g)**	Overweight & low stress n=16	-0.0024	0.0075	0.7476		
	Normal weight & high stress n=45	0.003	0.0047	0.5333		
	Overweight & high stress n=23	0.0025	0.0078	0.7439		
	Total model			0.8217	0.0068	0.2251
**HFSW intake (kcal)**	Overweight & low stress n=16	-0.0626	1.3736	0.9638		
	Normal weight & high stress n=45	0.4284	0.8616	0.6200		
	Overweight & high stress n=23	2.2846	1.4205	0.1108		
	Total model			0.4086	0.0229	0.1762
**LFSW intake (kcal)**	Overweight & low stress n=16	1.3735	0.7864	0.0837		
	Normal weight & high stress n=45	0.5782	0.4933	0.2438		
	Overweight & high stress n=23	0.2209	0.8133	0.7864		

	Total model			0.3387	0.0282	0.1302
**HFSA intake (kcal)**	Overweight & low stress n=16	0.0006	1.5277	0.9997		
	Normal weight & high stress n=45	0.5614	0.9399	0.5517		
	Overweight & high stress n=23	0.6936	1.545	0.6544		
	Total model			0.9178	0.0045	0.1016
**LFSA intake (kcal)**	Overweight & low stress n=16	-0.1113	0.3876	0.7746		
	Normal weight & high stress n=45	-0.3645	0.2434	0.1374		
	Overweight & high stress n=23	-0.1647	0.3922	0.6754		
	Total model			0.4646	0.0252	0.0522
		**% Stress index recovery**				
**Food lab responses**		**b_{int}**	**SE_{int}**	**p_{int}**	**ΔR^2_{int}**	**R^2**
**Food intake (kcal)**	Overweight & low stress n=16	-4.3186	3.57	0.2291		
	Normal weight & high stress n=45	-5.9372	2.408	0.0153		
	Overweight & high stress n=23	-1.5027	3.3324	0.6530		
	Total model			0.0880	0.0478	0.2529
**Energy density intake (kcal/g)**	Overweight & low stress n=16	0.0187	0.0093	0.0474		
	Normal weight & high stress n=45	-0.0005	0.0063	0.9305		
	Overweight & high stress n=23	0.0056	0.0087	0.5174		
	Total model			0.1539	0.0376	0.2649
**HFSW intake (kcal)**	Overweight & low stress n=16	-0.5653	1.6094	0.7261		
	Normal weight & high stress n=45	-2.3583	1.0856	0.0321		
	Overweight & high stress n=23	0.6664	1.5023	0.6583		
	Total model			0.0637	0.0501	0.2984
**LFSW intake (kcal)**	Overweight & low stress n=16	-1.5123	0.9774	0.1248		
	Normal weight & high stress n=45	-1.4216	0.6593	0.0333		
	Overweight & high stress n=23	-1.282	0.9124	0.1629		
	Total model			0.1610	0.0417	0.1666
**HFSA intake (kcal)**	Overweight & low stress n=16	-0.3321	0.343	0.3353		
	Normal weight & high stress n=45	-0.2583	0.2353	0.2748		
	Overweight & high stress n=23	-0.0328	0.3176	0.9179		
	Total model			0.6210	0.0157	0.1199
**LFSA intake (kcal)**	Overweight & low stress n=16	-0.6829	0.4751	0.1538		

	Normal weight & high stress n=45	-0.5924	0.3366	0.0816		
	Overweight & high stress n=23	-0.8307	0.4357	0.0595		
	Total model			0.1796	0.0458	0.1114
		**AUCi Stress index**				
**Food lab responses**		**b_{int}**	**SE_{int}**	**p_{int}**	**ΔR^2_{int}**	**R^2**
**Food intake (kcal)**	Overweight & low stress n=16	0.1068	2.9711	0.9714		
	Normal weight & high stress n=45	-2.2977	2.0047	0.2543		
	Overweight & high stress n=23	2.7856	3.2502	0.3934		
	Total model			0.2872	0.0298	0.1815
**Energy density intake (kcal/g)**	Overweight & low stress n=16	0.0003	0.0076	0.9718		
	Normal weight & high stress n=45	-0.0006	0.0051	0.9072		
	Overweight & high stress n=23	0.0067	0.0083	0.4201		
	Total model			0.8180	0.0069	0.2237
**HFSW intake (kcal)**	Overweight & low stress n=16	-0.3867	1.3593	0.7766		
	Normal weight & high stress n=45	-0.9849	0.9172	0.2853		
	Overweight & high stress n=23	2.5192	1.487	0.0932		
	Total model			0.0797	0.0524	0.2084
**LFSW intake (kcal)**	Overweight & low stress n=16	-0.1174	0.5341	0.8264		
	Normal weight & high stress n=45	-0.1085	0.866	0.9006		
	Overweight & high stress n=23	0.1419	0.2653	0.5938		
	Total model			0.5981	0.0155	0.1353
**HFSA intake (kcal)**	Overweight & low stress n=16	-0.0496	1.5262	0.9741		
	Normal weight & high stress n=45	-0.4886	1.028	0.6356		
	Overweight & high stress n=23	0.7165	1.644	0.6639		
	Total model			0.8614	0.0067	0.1086
**LFSA intake (kcal)**	Overweight & low stress n=16	-0.2354	0.3777	0.5345		
	Normal weight & high stress n=45	-0.4896	0.2595	0.0622		
	Overweight & high stress n=23	-0.3379	0.4092	0.4109		
	Total model			0.6115	0.0939	0.0339
		**% RMSSD reactivity**				
**Food lab responses**		**b_{int}**	**SE_{int}**	**p_{int}**	**ΔR^2_{int}**	**R^2**
**Food intake (kcal)**	Overweight & low stress n=16	-0.0237	2.8034	0.9933		

	Normal weight & high stress n=45	-1.3337	1.5134	0.3802		
	Overweight & high stress n=23	-2.8561	2.8217	0.3138		
	Total model			0.6945	0.0117	0.1525
**Energy density intake (kcal/g)**	Overweight & low stress n=16	0.0090	0.0070	0.2010		
	Normal weight & high stress n=45	0.0011	0.0038	0.7724		
	Overweight & high stress n=23	-0.0024	0.0071	0.7363		
	Total model			0.5457	0.0157	0.2328
**HFSW intake (kcal)**	Overweight & low stress n=16	-0.2142	1.2886	0.8683		
	Normal weight & high stress n=45	-0.8203	0.6956	0.2410		
	Overweight & high stress n=23	-1.9714	1.2970	0.1315		
	Total model			0.4285	0.022	0.1727
**LFSW intake (kcal)**	Overweight & low stress n=16	-0.7571	0.7434	0.3108		
	Normal weight & high stress n=45	-0.3263	0.4013	0.4180		
	Overweight & high stress n=23	-0.2424	0.7482	0.7466		
	Total model			0.7507	0.0102	0.1132
**HFSA intake (kcal)**	Overweight & low stress n=16	0.8965	1.4375	0.5343		
	Normal weight & high stress n=45	-0.334	0.7566	0.6599		
	Overweight & high stress n=23	-0.5832	1.4065	0.6793		
	Total model			0.7852	0.0095	0.1059
**LFSA intake (kcal)**	Overweight & low stress n=16	-0.0607	0.3592	0.8663		
	Normal weight & high stress n=45	0.097	0.2	0.6286		
	Overweight & high stress n=23	-0.1203	0.3613	0.7398		
	Total model			0.8421	0.0082	0.044
		**% RMSSD recovery**				
**Food lab responses**		**b_{int}**	**SE_{int}**	**p_{int}**	**ΔR^2_{int}**	**R^2**
**Food intake (kcal)**	Overweight & low stress n=16	4.5226	4.2356	0.2881		
	Normal weight & high stress n=45	4.1126	1.9192	0.0344		
	Overweight & high stress n=23	3.3089	2.7614	0.2335		
	Total model			0.1978	0.0358	0.209
**Energy density intake (kcal/g)**	Overweight & low stress n=16	-0.018	0.0108	0.0999		
	Normal weight & high stress n=45	-0.0024	0.0049	0.6326		
	Overweight & high stress n=23	-0.0093	0.0071	0.1928		

	Total model			0.2668	0.0287	0.2489
**HFSW intake (kcal)**	Overweight & low stress n=16	0.314	1.9347	0.8714		
	Normal weight & high stress n=45	1.5229	0.8766	0.0853		
	Overweight & high stress n=23	0.3971	1.2613	0.7535		
	Total model			0.3147	0.0261	0.2375
**LFSW intake (kcal)**	Overweight & low stress n=16	2.317	1.1258	**0.0421**		
	Normal weight & high stress n=45	1.1888	0.5101	**0.0217**		
	Overweight & high stress n=23	1.5503	0.734	**0.0370**		
	Total model			**0.0463**	0.0654	0.1683
**HFSA intake (kcal)**	Overweight & low stress n=16	2.1115	2.2834	0.3574		
	Normal weight & high stress n=45	0.713	0.9915	0.4737		
	Overweight & high stress n=23	0.7253	1.4194	0.6105		
	Total model			0.7847	0.0095	0.1116
**LFSA intake (kcal)**	Overweight & low stress n=16	0.7076	0.5507	0.2018		
	Normal weight & high stress n=45	0.678	0.2571	**0.0098**		
	Overweight & high stress n=23	0.8185	0.3488	**0.0210**		
	Total model			**0.0431**	0.0755	0.133
		**AUCi RMSSD**				
**Food lab responses**		b_{int}	SE_{int}	p_{int}	ΔR^2_{int}	R^2
**Food intake (kcal)**	Overweight & low stress n=16	0.4604	0.4513	0.3100		
	Normal weight & high stress n=45	-0.0566	0.2034	0.7814		
	Overweight & high stress n=23	-0.1694	0.2859	0.5548		
	Total model			0.6062	0.0149	0.1557
**Energy density intake (kcal/g)**	Overweight & low stress n=16	0.0007	0.0011	0.5428		
	Normal weight & high stress n=45	-0.0001	0.0005	0.9085		
	Overweight & high stress n=23	-0.0001	0.0007	0.9295		
	Total model			0.9213	0.0036	0.2222
**HFSW intake (kcal)**	Overweight & low stress n=16	0.0414	0.2076	0.8423		
	Normal weight & high stress n=45	-0.0441	0.0936	0.6386		
	Overweight & high stress n=23	-0.1994	0.1315	0.1327		
	Total model			0.4270	0.022	0.1745
**LFSW intake (kcal)**	Overweight & low stress n=16	0.0577	0.1200	0.6314		

	Normal weight & high stress n=45	0.0132	0.0541	0.8075		
	Overweight & high stress n=23	0.0737	0.0760	0.3345		
	Total model			0.7510	0.0102	0.1113
**HFSA intake (kcal)**	Overweight & low stress n=16	0.4552	0.2313	0.0518		
	Normal weight & high stress n=45	-0.0843	0.0991	0.3972		
	Overweight & high stress n=23	-0.0839	0.139	0.5476		
	Total model			0.1027	0.0538	0.1526
**LFSA intake (kcal)**	Overweight & low stress n=16	0.0252	0.0593	0.6724		
	Normal weight & high stress n=45	0.058	0.0255	0.0251		
	Overweight & high stress n=23	0.0473	0.0357	0.1882		
	Total model			0.1614	0.0495	0.0862

Values given are in comparison to the control group: Low Stress Normal Weight (n=52). Significance is visualized by bolded for p≤0.05. Linear regression estimated marginal means and standard errors (mean±SE) adjusted for age, sex and parental education, hunger at study start and liking of presented food. AUCi: area under the curve with respect to increase; RMSSD: Root Mean Square of the Successive Difference; HF: high fat; LF: low fat; SW: sweet; SA: savoury
