# Supplementary material for: Stress Responsiveness and Emotional Eating Depend on Youngsters’ Chronic Stress Level and Overweight
Source: Nutrients. 2021 Oct 19;13(10):3654. doi: 10.3390/nu13103654 (PMC8540677; doi:10.3390/nu13103654)
Supplement: Supplementary file 1 [file nutrients-13-03654-s001.zip › Table S6.pdf]

Table S6. Relations between continuous stress reactivity/recovery/AUCi and food lab responses after the Trier Social Stress Test in adolescents.

		**Total food intake**		**HFSW intake**	**LFSW intake**	**HFSA intake**	**LFSA intake**	**Wanting**
		(kcal)	(kcal/g)	(kcal)	(kcal)	(kcal)	(kcal)	**increase**
**% Salivary alpha-amylase reactivity**	β	-0.013	0.072	0.008	-0.064	0.000	-0.107	-0.033
	p	0.880	0.399	0.926	0.468	0.997	0.263	0.707
**% Salivary alpha-amylase recovery**	β	-0.045	-0.112	-0.090	0.007	0.030	-0.139	0.130
	p	0.620	0.207	0.327	0.938	0.754	0.158	0.181
**AUCi Salivary alpha-amylase**	β	-0.021	0.011	0.045	0.020	-0.064	-0.180	0.001
	p	0.812	0.900	0.608	0.823	0.486	0.057	0.992
**% Salivary cortisol reactivity**	β	0.101	0.048	0.223	-0.026	-0.031	0.033	-0.118
	p	0.285	0.604	**0.018**	0.785	0.759	0.753	0.221
**% Salivary cortisol recovery**	β	-0.061	0.093	-0.023	0.020	-0.087	-0.040	0.020
	p	0.557	0.376	0.822	0.848	0.428	0.733	0.850
**AUCi Salivary cortisol**	β	0.112	0.125	0.049	0.120	0.108	0.077	0.050
	p	0.204	0.147	0.581	0.175	0.247	0.425	0.583
**% Stress increase**	β	-0.016	0.010	0.098	-0.030	-0.118	-0.021	-0.265
	p	0.857	0.904	0.268	0.731	0.200	0.831	0.003
**% Stress recovery**	β	0.064	0.135	0.077	0.023	0.054	-0.023	0.173
	p	0.461	0.114	0.382	0.792	0.555	0.813	**0.050**
**AUCi Stress**	β	0.026	0.092	-0.035	0.034	0.030	0.112	0.079
	p	0.771	0.287	0.695	0.705	0.747	0.251	0.379
**% Negative emotions reactivity**	β	0.058	0.054	-0.002	0.038	0.151	0.070	0.030
	p	0.503	0.524	0.982	0.665	0.090	0.457	0.738
**% Negative emotions recovery**	β	0.131	0.147	0.126	-0.020	0.164	-0.031	0.022
	p	0.139	0.095	0.163	0.827	0.080	0.760	0.812
**AUCi Negative emotions**	β	0.125	0.013	0.119	0.103	0.085	0.016	0.044
	p	0.153	0.879	0.179	0.247	0.350	0.600	0.617
**% Happy reactivity**	β	0.123	0.121	0.127	-0.027	0.152	-0.059	-0.043
	p	0.156	0.158	0.149	0.760	0.094	0.537	0.624
**% Happy recovery**	β	-0.042	0.001	0.001	-0.048	-0.074	0.013	0.277
	p	0.626	0.986	0.991	0.580	0.409	0.887	0.001
**AUCi happy**	β	0.110	0.039	0.147	-0.038	0.071	-0.047	-0.148
	p	0.201	0.642	0.091	0.667	0.430	0.620	0.089
**% Stress index reactivity**	β	-0.038	-0.060	-0.045	0.026	-0.037	0.018	-0.055
	p	0.685	0.492	0.635	0.788	0.703	0.861	0.559
**% Stress index recovery**	β	0.245	0.099	0.317	0.132	0.101	0.244	0.173
	p	**0.015**	0.305	**0.002**	0.203	0.347	**0.024**	0.092
**AUCi Stress index**	β	0.056	-0.059	0.046	0.064	0.067	0.149	0.054
	p	0.565	0.525	0.639	0.641	0.512	0.167	0.581
**% RMSSD reactivity**	β	-0.025	0.043	0.008	-0.057	-0.042	-0.067	-0.019
	p	0.787	0.625	0.932	0.542	0.665	0.509	0.841
**% RMSSD recovery**	β	-0.210	-0.070	-0.263	-0.068	-0.109	-0.247	-0.272
	p	**0.031**	0.453	**0.007**	0.499	0.284	**0.019**	**0.005**
**AUCi RMSSD**	β	-0.012	-0.059	-0.059	-0.018	0.054	-0.090	0.009
	P	0.897	0.518	0.548	0.852	0.593	0.395	0.926

Standardized regression coefficients from linear regressions adjusted for age, sex and parental education, hunger at study start and liking of presented snacks. All recovery analyses were additionally adjusted for their corresponding % reactivity. AUCi: area under the curve with respect to increase; RMSSD: Root Mean Square of the Successive Differences.
