# Supplementary material for: Stress Responsiveness and Emotional Eating Depend on Youngsters’ Chronic Stress Level and Overweight
Source: Nutrients. 2021 Oct 19;13(10):3654. doi: 10.3390/nu13103654 (PMC8540677; doi:10.3390/nu13103654)
Supplement: Supplementary file 1 [file nutrients-13-03654-s001.zip › Table S7.pdf]

Table S7. Linear regression with continuous trait emotional eating as predictor and state emotional eating as outcome

	Trait emotional eating	
	β	p
Increase in hunger	-0.105	0.257
Increase in wanting	-0.108	0.233
Food intake (kcal)	0.060	0.499
Energy density intake (kcal/g)	0.112	0.204
HFSW intake (kcal)	0.067	0.460
LFSW intake (kcal)	0.024	0.787
HFSA intake (kcal)	0.070	0.458
LFSA intake (kcal)	-0.101	0.303

Linear regression with continuous trait emotional eating as predictor and state emotional eating as outcome. Standardized regression coefficients were adjusted for age, gender and parental education, hunger at study start and liking of presented food. HF: high fat; LF: low fat; SW: sweet; SA: savoury.
